# Incidence and impact of brain metastasis in patients with hereditary *BRCA1 or BRCA2* mutated invasive breast cancer

**DOI:** 10.1038/s41523-022-00407-z

**Published:** 2022-04-07

**Authors:** Haven R. Garber, Akshara Singareeka Raghavendra, Michael Lehner, Wei Qiao, Angelica M. Gutierrez-Barrera, Debu Tripathy, Banu Arun, Nuhad K. Ibrahim

**Affiliations:** 1 Department of Breast Medical Oncology, University of Texas MD Anderson Cancer Center, 1515 Holcombe Boulevard, Houston, TX 77030 USA; 2 Departments of UT Internal Medicine Residency Training Program, University of Texas MD Anderson Cancer Center, 1515 Holcombe Boulevard, Houston, TX 77030 USA; 3 Departments of Biostatistics, University of Texas MD Anderson Cancer Center, 1515 Holcombe Boulevard, Houston, TX 77030 USA

**Keywords:** Breast cancer, Cancer genetics

## Abstract

Patients with hereditary mutations in *BRCA1* or *BRCA2 (gBRCA1/2)* and breast cancer have distinct tumor biology, and encompass a predilection for brain metastasis (BM). We looked into baseline risk of BMs among *gBRCA1/2* patients. Patients with *gBRCA1/2*, stage I-III invasive breast cancer seen between 2000–2017 with parenchymal BMs. Among *gBRCA1* with distant breast cancer recurrence, 34 of 76 (44.7%) were diagnosed with brain metastases compared to 7 of 42 (16.7%) patients with *gBRCA2*. In the comparator group, 65 of 182 (35.7%) noncarrier triple-negative breast cancer (TNBC) and a distant recurrence experienced BM’s. In a competitive risk analysis using death as a competing factor, the cumulative incidence of BMs was similar between *gBRCA1* and noncarrier TNBC patients. The time from primary breast cancer diagnosis to detection of BMs was similar between *gBRCA1* and noncarrier TNBC patients (2.4 vs 2.2 years). Survival was poor after BMs (7.8 months for *gBRCA1* patients vs. 6.2 months for TNBC noncarriers). Brain was a more common site of initial distant recurrence in *gBRCA1* patients versus TNBC noncarriers (26.3% vs. 12.1%). Importantly, the presence of BMs, adversely impacted overall survival across groups (HR 1.68 (95% CI 1.12–2.53), hazard ratio for death if a patient had BMs at the time of initial breast cancer recurrence vs. not). In conclusion, breast cancer BMs is common and is similarly frequent among *gBRCA1* and noncarrier patients with recurrent TNBC. Our study highlights the importance of improving the prevention and treatment of BMs in patients with TNBC, *gBRCA1* carriers, and noncarriers.

## Introduction

Hereditary pathogenic variants in the *BRCA1* and *BRCA2* genes substantially increase the risk of developing breast cancer, ovarian cancer, and other malignancies^1^. Pathogenic variants in either gene account for ~40% of hereditary breast and ovarian cancers and ~5% of total breast cancers^2^. Both genes function in the repair of double-strand DNA breaks through homologous recombination and operate as tumor suppressor genes. Hereditary pathogenic variants are typically heterozygous loss-of-function alterations^3^. The breast cancer phenotype associated with germline *BRCA* pathogenic variants (*gBRCA*) differs between *gBRCA1* and *gBRCA2*, reflecting their disparate roles in homologous recombination. Breast cancer-associated with *gBRCA1* is far more likely to be triple-negative breast cancer (TNBC), an aggressive subtype associated with a poor prognosis, whereas *gBRCA2* breast cancers exhibit a similar receptor subtype distribution to sporadic breast cancers^4^.

Genetic testing for *gBRCA* is recommended for patients with metastatic breast cancer in whom it could help guide systemic therapy and for patients with any stage breast cancer who are considered to be at a high risk for hereditary breast cancer^5^. Genetic testing results inform breast cancer screening, risk reduction strategies, and family counseling, and they are now integral to the treatment of metastatic breast cancer. In 2018, the FDA approved the Poly (ADP-ribose) polymerase (PARP) inhibitors olaparib and talazoparib for the treatment of HER2-negative, *gBRCA*-associated metastatic breast cancer. Both PARP inhibitors proved superior to chemotherapy in terms of progression-free survival (PFS) in phase III clinical trials that included metastatic breast cancer patients with *gBRCA*^6,7^. In the EMBRACA trial that evaluated talazoparib, ~15% of patients had brain metastases; however, to be eligible, patients with central nervous system (CNS) disease had to receive definitive local CNS therapy prior to the study. The investigators reported a comparable benefit of talazoparib in this subgroup of patients compared to the total population, though granular data on responses in the CNS were not provided in the initial report^6^. The combination of veliparib, carboplatin, and paclitaxel also demonstrated superior PFS versus placebo/carboplatin/paclitaxel in the Phase III BROCADE3 trial that included 509 patients with *gBRCA1/2* and HER2-negative advanced breast cancer. In BROCADE3, patients with CNS disease composed ~5% of those enrolled and so they were not included as a separate subgroup^8^.

The prospect of improved systemic disease control with PARP inhibitors and emerging related therapies led us to ask whether we might observe more frequent CNS metastases among patients with *gBRCA* in the future, particularly if a propensity for brain metastasis exists in this patient subgroup^9–12^. This phenomenon has been observed in patients with metastatic HER2-positive breast cancer as tremendous advances have been made in treating systemic disease but effective treatments for CNS metastasis have lagged. Recently, clinical trials allowing enrollment of patients with untreated brain metastases have led to the approval of drugs like tucatinib, which demonstrated clear CNS activity^13–15^. Data regarding PARP inhibitor penetration of the blood-brain barrier (BBB) are mixed and likely relate to the extent and location of CNS metastases and the associated disruption of the BBB^16–18^.

The goal of this study was to determine the incidence of brain metastasis in *gBRCA* patients presenting with early-stage breast cancer. We also sought to determine the impact of brain metastasis on survival in patients with *gBRCA* and noncarriers with recurrent metastatic breast cancer. A high incidence of brain metastasis in *gBRCA* patients could provide rationale for prioritizing therapeutic agents with CNS activity, considering brain MRIs at the time of distant recurrence, and studying the biologic mechanisms of *gBRCA*-mediated CNS metastasis.

## Results

**Fig. 1:**
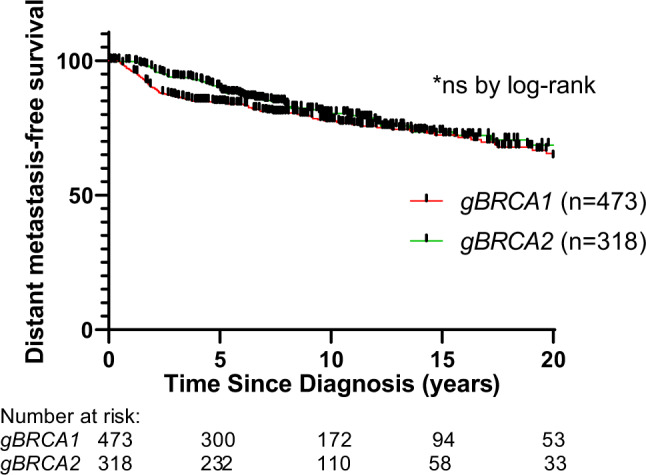
Distant metastasis-free survival. Distant metastasis-free survival for patients diagnosed with stage I-III breast cancer by gBRCA status.

Between 2000 and 2017, we identified 473 patients with *gBRCA1* and 318 patients with *gBRCA2* who were evaluated for stage I-III breast cancer at MDACC and who were captured in the clinical cancer genetics database. The median length of follow-up for the *gBRCA1* and *gBRCA2* patients was 9.15 years and 8.45 years, respectively. A total of 76 of 473 (16.1%) *gBRCA1* patients and 42 of 318 (13.2%) *gBRCA2* patients experienced a distant metastasis from breast cancer (Fig. 1). At 3 years, distant metastasis-free survival was inferior in *gBRCA1* patients compared to *gBRCA2* patients (86.7%) (95% confidence interval [CI], 83.2–90) vs. 94% (95% CI, 90.6–96.1); however, the Kaplan–Meier curves did not differ significantly overall (*P* = 0.25 by log-rank).

Brain metastases were diagnosed in 34 of 76 (44.7%) *gBRCA1* patients with distantly recurrent disease and in 7 of 42 (16.7%) *gBRCA2* patients with distantly recurrent disease. As expected, the *gBRCA1* patients with distant metastasis had predominantly TNBC (77.6%, Table 1) whereas *gBRCA2* patients had primarily HR-positive/HER2-negative disease (71.4%). Of the 34 *gBRCA1* patients with eventual brain metastasis, 33 had TNBC, and 1 had HR-positive/HER2-negative primary breast cancer, though this patient’s disease lost ER/PR expression at the time of distant recurrence. Since the *gBRCA1* cohort had the higher incidence of brain metastases and primarily TNBC, *gBRCA* noncarriers with TNBC and distant metastasis were utilized as the comparator group.

**Table 1:** Clinical characteristics of patients with distantly recurrent breast cancer by germline *BRCA* status.

	*BRCA Status*						
	germline *BRCA1* pathogenic variant (*n* = 76)		germline *BRCA2* pathogenic variant (*n* = 42)		*BRCA1/2* non-carrier with TNBC (*n* = 182)		*P*
Clinical characteristic							
Median age at diagnosis (range)	38 (23–72)		44.8 (27–76)		47 (19–79)		<0.0001
Median time from diagnosis to hereditary genetic testing (IQR) in months	3.1 (0.8–16)		10.3 (1.2–114.1)		8.1 (1.8–28.1)		0.029
	No. of Patients	%	No. of Patients	%	No. of Patients	%	
Sex							
Female	76	100	39	92.9	182	100	
Male			3	7.1			
Race							
White	41	53.9	32	76.2	118	64.8	<0.0001
Black	15	19.7	3	7.1	31	17.0	
Hispanic	13	17.1	3	7.1	24	13.2	
Asian/Pacific Islander	5	6.6	4	9.5	6	3.3	
Other	2	2.6			3	1.6	
Breast cancer subtype*							
TNBC	59	77.6	4	9.5	178	97.8	<0.0001
HR-positive/HER2-negative	8	10.5	30	71.4			
HER2-positive	3	3.9	5	11.9			
HR-positive/HER2 unknown	2	2.6	3	7.1			
HR-negative/HER2 unknown	2	2.6			4	2.2	
Unknown	2	2.6					
2nd primary breast cancer	5	6.6	5	11.9	13	7.1	0.532
Stage (anatomic)							
1	16	21.1	11	26.2	18	9.9	0.027
2	29	38.2	18	42.9	91	50.0	
3	29	38.2	13	31.0	73	40.1	
Unknown	2	2.6					
Tumor grade							
1	3	3.9			2	1.1	<0.0001
2	12	15.8	20	47.6	18	9.9	
3	56	73.7	20	47.6	157	86.3	
Unknown	5	6.6	2	4.8	5	2.7	
Histology							
IDC	71	93.4	36	85.7	163	89.6	<0.0001
ILC	1	1.3	5 (mixed IDC/ILC)	11.9	4 (3 are mixed IDC/ILC)	2.2	
Other or breast cancer, NOS	3	3.9	1	2.4	15	8.2	
Unknown	1	1.3					

*For patients with a second primary breast cancer, the most recent subtype is listed.

Breast cancer was diagnosed at a younger age in *gBRCA1* patients compared to *gBRCA2* patients and noncarriers (median age 38 vs. 44.8 vs. 47, *P* < 0.0001). The median time to genetic testing was shorter in *gBRCA1* patients than in *gBRCA2* patients and noncarriers (median 3.1 vs. 10.3 vs. 8.1 months, *P* = 0.029). Diagnosis of a second primary breast tumor occurred in 5 of 76 (6.5%) *gBRCA1* patients, 5 of 42 (11.9%) of *gBRCA2* patients, and 13 of 182 (7.1%) of noncarriers. Additional patient and disease characteristics including patient race, cancer stage, tumor grade, and tumor histology are listed in Table 1.

Most patients had a mastectomy for surgical resection of the primary breast tumor, though approximately 20% or more of patients in each group had a partial mastectomy (Table 2). Systemic chemotherapy was administered for the primary breast cancer in the majority (>80%) of cases. More than half of patients in the *gBRCA1* and noncarrier groups received neoadjuvant chemotherapy, reflecting the preference for upfront chemotherapy for TNBC. Chemotherapy was largely anthracycline-based across groups.

**Table 2:** Treatment characteristics of patients with distantly recurrent breast cancer by germline *BRCA* status.

	*BRCA Status*						
	germline *BRCA1* pathogenic variant (*n* = 76)		germline *BRCA2* pathogenic variant (*n* = 42)		*BRCA1/2* noncarrier with TNBC (*n* = 182)		*P*
	No. of Patients	%	No. of Patients	%	No. of Patients	%	
Treatment characteristic*							
**Surgery type**							
Partial mastectomy	16	21	8	19	66	36	0.04
Mastectomy	57	75	34	81	112	62	
ALND only for occult breast primary	0	0	0	0	2	1	
Unknown	3	4	0	0	2	1	
**Chemotherapy**							
Neoadjuvant	40	53	15	36	97	53	0.0001
Adjuvant	13	17	16	38	66	36	
No chemotherapy	14	18	7	17	5	3	
Neoadjuvant + adjuvant	9	12	4	10	14	8	
**Chemotherapy regimen**							
Anthracycline-based (non-taxane)^#^	12	17	8	21	27	14	
Anthracycline + taxane	46	65	23	59	135	71	
Taxane based	8	11	6	15	23	12	
else	5	7	2	5	6	3	
**Radiation therapy**							
Yes	47	62	30	71	126	69	0.677
No	26	34	12	29	56	31	
Unknown	3	4	0	0	0	0	

*For patients with a second primary breast cancer, the treatment for the most recent breast cancer is listed.

^#^These patients primarily received FAC chemotherapy.

For the 106 patients who eventually developed brain metastases, the median time to the detection of brain metastases from the initial breast cancer diagnosis was similar between *gBRCA1* patients and noncarriers (2.4 and 2.2 years) and was longer for *gBRCA2* patients (5 years), though this was not statistically significant owing to the small size of the *gBRCA2* cohort (Table 3). Among the 7 *gBRCA2* patients who developed eventual brain metastases, all were female, 2 patients had HER2+ disease, and the remaining 5 patients had HR+/HER2-negative disease. All 7 patients received adjuvant chemotherapy for the primary breast tumor and 6 of 7 (85.7%) received adjuvant radiation. Due to the small number of *gBRCA2* patients with brain metastases, we focused on estimating the cumulative incidence of brain metastasis among *gBRCA1* patients and noncarriers with a distant recurrence.

**Table 3:** Characteristics of brain metastases in patients with distantly recurrent breast cancer by germline BRCA status.

	*BRCA Status*						
	germline *BRCA1* pathogenic variant (*n* = 76)		germline *BRCA2* pathogenic variant (*n* = 42)		*BRCA1/2* noncarrier with TNBC (*n* = 182)		*P*
	No. of Patients	%	No. of Patients	%	No. of Patients	%	
Breast cancer brain metastases detected at any time during patient’s course							
Yes	34	44.7	7	16.7	65	35.7	0.009
No	42	55.3	35	83.3	117	64.3	
Median time to detection of brain metastases (years from date of diagnosis, range)	2.4 (0.7–31.9)		4.98 (1.7–14.4)		2.2 (0.5–6.5)		0.12
Was the brain a site of initial distant recurrence?							
Yes	20	26.3	3	7.1	22	12.1	0.058
No, brain mets were found later in course	14	18.4	4	9.5	43	23.6	
No brain mets	42	55.3	35	83.3	117	64.3	
If brain mets were among the initial site(s) of distant recurrence, was extracranial disease absent or present at the time of recurrence?							0.53
Extracranial disease absent	11		1		9		0.587
Extracranial disease present	9		2		13		
Number of brain metastases at time of initial detection (as a %age of patients w/brain metastasis)							
solitary	12	35.3	1	14.3	21	32.3	0.2501
2–3 brain metastases	8	23.5			11	16.9	
>3 brain metastases	13	38.2	6	85.7	32	49.2	
unknown	1	2.9			1	1.5	
Treatment for initial brain metastases*							
WBRT	22	56.4	4	50	36	48.6	
SRS/SRT	8	20.5			25	33.8	
Resection	8	20.5	2	25	10	13.5	
Hospice	1	2.6	2	25	3	4.1	
Unknown							

*Many patients were treated with multimodality therapy (e.g., resection followed by either SRS or WBRT). All treatments administered for the patients’ initial presentation of brain metastases are listed.

**Fig. 2:**
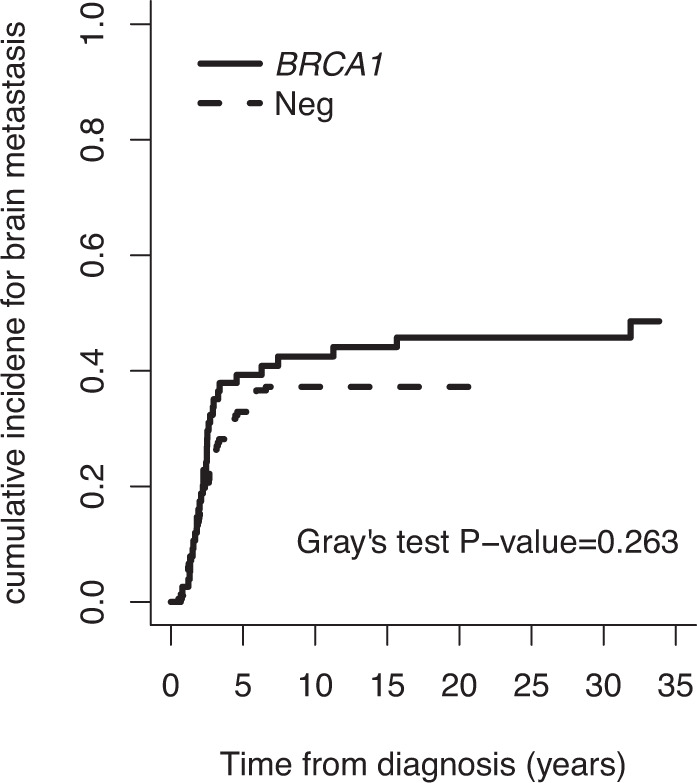
Cumulative incidence of brain metastasis. Estimates of the cumulative incidence of brain metastasis are shown for gBRCA1 patients (*n* = 76) and noncarriers with triple negative breast cancer (*n* = 182) diagnosed with stage I-III disease with subsequent distant recurrence.

Using death as a competing risk factor, the cumulative incidence of brain metastasis for *gBRCA1* patients at 2 and 5 years from initial diagnosis was 17.2% (95% CI = 9.7–26.5%) and 38.8% (95% CI = 27.7–49.1%), respectively, compared to 15.4% (95% CI = 10.6–21.1%) and 33.3% (95% CI = 26.4–40.3%) for noncarriers (Fig. 2). In the competitive risk analysis, the cumulative incidence of brain metastasis was not different between the *gBRCA1* and noncarrier cohorts (*P* = 0.263 by Gray’s test).

**Fig. 3:**
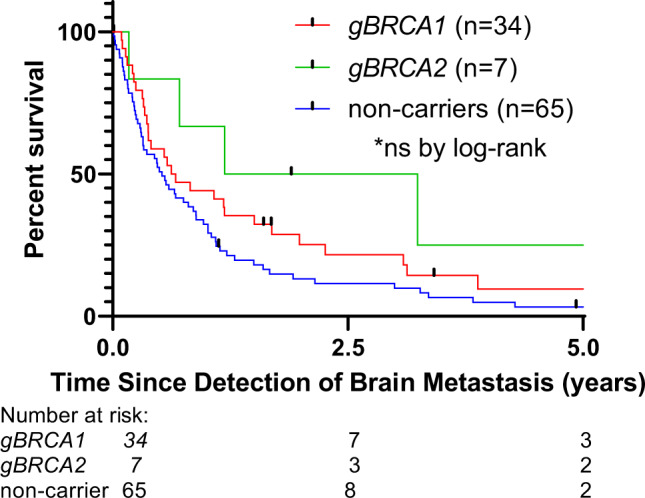
Overall Survival with brain metastasis. Overall survival from the time of brain metastasis detection for breast cancer patients by gBRCA status.

The brain parenchyma was a more frequent site of initial recurrence in *gBRCA1* patients versus noncarriers (20/76 [26.3%] vs. 22/182 [12.1%], *P* = .01 by Fisher’s exact test between the two groups). Brain metastases were diagnosed as the initial recurrence in the absence of extracranial disease (i.e., isolated brain parenchymal metastases) in 11 of 20 *gBRCA1* patients and 9 of 22 noncarriers. Among patients with brain metastasis, approximately one-third of *gBRCA1* and noncarrier patients had a solitary brain metastasis whereas 13 of 34 (38.2%) *gBRCA1* patients, 6 of 7 (85.7%) of *gBRCA2* patients, and 32 of 65 (49.2%) noncarriers had more than three brain metastases on the initial brain MRI. The treatments administered for brain metastasis included whole-brain radiotherapy (WBRT), stereotactic radiosurgery or radiotherapy (SRS/SRT), and surgical resection. There were no large differences in the modalities selected for *gBRCA1* patients versus noncarriers. The median overall survival (OS) from the time of brain metastasis detection was uniformly poor: 7.8 months in *gBRCA1* patients, 26.6 months in *gBRCA2* patients, and 6.2 months in noncarriers (Fig. 3).

**Fig. 4:**
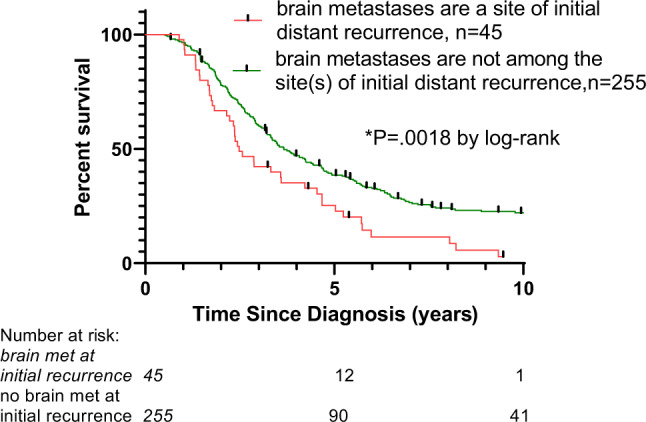
Overall Survival with distant recurrence. Overall survival from the time of diagnosis for patients with recurrent breast cancer stratified by the presence or absence of brain metastases among the initial sites of disease recurrence.

The presence of brain metastasis, irrespective of its timing, adversely impacted OS (Fig. 4 and Supplementary Figure 1). For example, the 45 patients who had brain metastases detected at the time of first recurrence had a median OS of 2.5 years compared to 3.4 years for patients who did not have brain metastases at the time of initial recurrence (HR 1.68, 95% CI 1.12–2.53). A similar trend was seen within the *gBRCA1* cohort (Supplementary Figure 2).

Lastly, we assessed potential clinical predictors of brain metastasis among *gBRCA1* and noncarrier patients in a multivariable logistic regression model. Univariate analyses were performed and variables with *P* ≤ .1 (breast cancer subtype, presence of a second primary breast tumor, tumor grade, adjuvant radiation) were then included in a multivariable model (Table 4; Supplementary Tables 1a/1b). In the multivariable model, TNBC was associated with higher rates of brain metastasis versus other breast cancer subtypes, which was expected and biased by our inclusion of noncarriers with TNBC only. Importantly, the model produced similar results with the breast cancer subtype variable removed (Supplemental Table 2). In addition, the odds ratio for brain metastasis among patients having received prior adjuvant radiation for a primary breast cancer versus no adjuvant radiation was 2.3 (95% CI 1.26–4.19, *P* = .0066).

**Table 4:** Multivariable logistic regression of potential clinical factors in brain metastasis (*n* = 258 *gBRCA1* and non-carrier patients).

Effect	Odds Ratio Estimates	95% Wald		
		Confidence Limits		*p*-value
Tumor grade (III vs. I/II)	2.425	0.971	6.055	0.0579
Breast ca subtype (others vs TNBC)	0.082	0.01	0.636	0.0167
Presence of a second primary tumor	0.25	0.053	1.186	0.081
Adj radiation (Yes vs. no)	2.296	1.261	4.181	0.0066

## Discussion

Our study of breast cancer patients with hereditary pathogenic variants in *gBRCA1* or *gBRCA2* revealed a high frequency of brain metastasis among women with distantly recurrent breast cancer and *gBRCA1*. There was no difference in predilection to extra cranial metastasis between *gBRCA1* or *gBRCA2* but revealed less incidence and longer time to brain metastasis of the tumors that are *gBRCA2* mutated compared to those that are *gBRCA1* mutated or non *BRCA* mutated TN breast cancer tumors. Among *gBRCA1* patients diagnosed with stage I-III breast cancer, 76 of 473 (16%) patients experienced a distant recurrence, and 34 of these 76 (44.7%) patients were diagnosed with brain metastases. The frequency of brain metastasis among *gBRCA2* patients with a distant recurrence was lower, with brain metastases detected in 7 of 42 (16.7%) *gBRCA2* patients in our cohort. As a comparator group for the *gBRCA1* cohort, we utilized patients diagnosed with stage I-III TNBC with recurrent breast cancer who had tested negative for variants in *BRCA1* and *BRCA2*. In a competitive risk analysis using death as a competing risk factor, the cumulative incidence of brain metastasis did not differ between *gBRCA1* patients and TNBC noncarrier patients. In addition, the time from primary breast cancer diagnosis to the detection of brain metastasis was similar between *gBRCA1* patients and TNBC noncarriers (2.4 vs. 2.2 years). One difference was that the brain was a more common site of initial distant recurrence in *gBRCA1* patients versus TNBC noncarriers (26.3% vs. 12.1%). In a multivariable logistic regression model, aside from TNBC subtype, only the receipt of prior adjuvant radiation (vs. no prior adjuvant radiation) for primary breast cancer treatment was associated with the eventual development of brain metastasis. We postulate that this is likely related to more aggressive primary tumors, those with a greater propensity to recur, being dispositioned to adjuvant radiation rather than due to untoward direct genotoxic effects from the radiation itself on residual tumor cells. Our multivariable model did not consider variables such as tumor stage/nodal stage (we included overall anatomic stage), proliferation index (Ki-67), margin status, and lymphovascular invasion, which may explain why the adjuvant radiation variable was significantly associated with brain metastasis in our model.

Patient survival after the detection of brain metastasis was expectedly poor across subgroups and was only 7.8 months for *gBRCA1* patients and 6.2 months in TNBC noncarriers. Survival after brain metastasis was longer in *gBRCA2* patients (2.2 years), though interpretation of these data is limited by the small cohort size (*n* = 7 patients). Brain metastasis had an adverse impact on survival in comparison to other sites of distant metastasis when considered as an initial event or at any time during the disease course.

We were interested in the baseline risk of brain metastasis among *gBRCA1* and *gBRCA2* patients in the pre-PARP inhibitor era to serve as context for evaluating CNS recurrences and responses now that PARP inhibitors and PARP inhibitors combinations are increasingly utilized in the adjuvant and metastatic settings^19^. PARP inhibitors improve systemic disease control and progression-free survival (an OS benefit has not been shown) versus standard chemotherapy, including in patients with treated brain metastases; however, their overall efficacy in the CNS remains uncertain. Two other institutions have reported their experience with *gBRCA* breast cancer patients and CNS recurrence. The Dana Farber Cancer Institute (DFCI)/Beth Israel Deaconess Medical Center (BIDMC) *gBRCA* cohort has been the focus of several important reports^10,11,20^. Lee et al. studied 46 *gBRCA1* and 71 *BRCA* noncarrier early-stage breast cancer patients who had received alkylating chemotherapy and found that the rate and distribution of distant metastasis and OS was similar between the two groups^20^. They reported a potential propensity for brain metastasis among *gBRCA1* patients (7 of 12 [58%] *gBRCA1* patients with brain metastasis vs. 5 of 21 (24%) noncarriers), which they examined in further detail in a follow-up report. This study included additional patients (a total of 89 *gBRCA1* and 175 noncarriers) and again compared the clinical outcomes of early-stage breast cancer patients treated with chemotherapy based on *gBRCA1* status, limiting the analyses to the TNBC subtype. No difference was observed between the two groups in terms of recurrence site, freedom from distant metastasis, or breast cancer-specific survival. The frequency of brain metastasis in *gBRCA1* patients with a distant breast cancer recurrence in this study was 7 of 19 (36.8%) patients and was 11 of 40 (27.5%) for noncarriers^11^. Our analysis of the MDACC cohort generally agrees with these data in demonstrating that the cumulative incidence of brain metastasis among *gBRCA1* and TNBC patients is similarly high. When we modified the competitive risk analysis to include only *gBRCA1* patients who had TNBC primary tumors (rather than other subtypes), the cumulative incidence of brain metastasis was significantly higher among *gBRCA1* TNBC patients vs. noncarrier TNBC patients (Supplementary Figure 3).

In a recent analysis of the DFCI *gBRCA* cohort, the authors reported a frequency of CNS metastasis (including parenchymal and leptomeningeal disease) among patients with recurrent breast cancer of 16/30 (53%) in *gBRCA1* patients, 16/32 (50%) in *gBRCA2* patients, and 67/270 (25%) in noncarriers (including all breast tumor subtypes, >50% HR+/HER2-negative)^10^. In a multivariable model within their study, only *gBRCA2* was significantly associated with CNS metastasis. The frequency of reported CNS metastasis in their *gBRCA2* cohort was higher than in ours [16/32 (50%) vs. 7/42 (16.7%)]. Both DFCI and MDACC are subject to referral bias though differences in the timing of referral may be one factor that explains the discrepancy. A second factor is that our primary event of interest was parenchymal brain metastasis rather than combined parenchymal brain metastasis/leptomeningeal disease. A third difference is that Song et al. relied on two databases, one that included only patients with recurrent breast cancer whereas we utilized one prospectively maintained database that included all patients referred for genetic testing. Lastly, the *gBRCA2* cohorts were limited in size in both studies. With regard to the multivariable model in Song et al. that showed *gBRCA2* as a risk factor for brain metastasis compared to noncarriers, our comparator group included only patients with TNBC, whereas *gBRCA2* breast cancer is predominantly HR+, and so our data do not address that specific question.

A smaller study from Gustave Roussy reported parenchymal brain metastasis among patients with distantly recurrent breast cancer in 10/15 (66.7%) *gBRCA1* patients, 0/12 *gBRCA2* patients, and 6/58 (10.3%) noncarriers (all breast tumor subtypes included)^9^. Taken together, these reports from the DFCI/BIDMC and Gustave Roussy along with our data from MDACC confirm a high rate of brain metastasis among *gBRCA1* patients and noncarriers with TNBC. There is no clear signal from the clinical data that *gBRCA* breast cancers have a unique predilection for the CNS, especially when compared to patients with TNBC, though it is plausible that different biologic mechanisms account for the high rate of CNS invasion in *gBRCA1* versus noncarrier TNBC recurrences. Investigators have studied brain metastasis in other BRCA-associated malignancies. Data from small cohorts suggest that *gBRCA* ovarian cancer patients may be at a higher risk for brain metastasis (or isolated brain metastasis), though the overall rate (~2.5%) is far lower than seen in breast cancer. Alternatively, those data may be explained by longer overall survival in *gBRCA* ovarian cancer patients attributable to enhanced chemosensitivity and effective PARP inhibitor maintenance therapy^21–26^. Brain metastasis in *gBRCA* pancreatic cancer patients is case reportable and is similarly rare in metastatic prostate cancer^27–29^.

Interestingly, increased homologous recombination deficiency (in wild-type *BRCA1/2* tumors) has been observed among breast cancer brain metastases^30,31^. In one study that examined the molecular features of paired primary breast tumors/brain metastases, an increase in homologous recombination deficiency was observed in the brain metastasis compared to the primary tumor in 14/16 (87.5%) of the cases^30^. Similar data have been reported for colorectal cancer brain metastases^32^. The investigators hypothesize that higher levels of homologous recombination deficiency may enable tumor cells to adapt more readily to the CNS microenvironment. In a systematic review of the genetic landscape of breast cancer brain metastases, several genes involved in *DNA* damage repair were among the 22 most frequently altered genes in brain metastases, including *BRCA1*, *BRCA2*, *MLH1*, *ATR*, *ATM*, and *CHEK2* whereas these genes were not among the most altered genes cataloged in extracranial distant recurrences^33–35^. Accordingly, two genes integral to homologous recombination, *BARD1*, and *RAD51*, were found to be overexpressed in breast cancer brain metastases compared to matched primary breast tumors, and when overexpressed in a human breast cancer cell line, these genes mediated increased brain metastases in mouse xenograft models^36^. No studies have focused exclusively on the molecular features of *gBRCA* brain metastases, and it would be interesting to determine whether these tumors undergo less genomic evolution upon CNS invasion since homologous recombination deficiency is intrinsic to the primary tumor. In addition, if TNBC tumors do become more BRCA-like upon seeding the brain, there is the possibility for therapeutic activity of PARP inhibitors with adequate CNS penetration. For example, it will be important to assess whether olaparib, as evaluated in the phase III OlympiA trial, was effective at preventing both systemic and CNS recurrences^19,37^.

Our study has several limitations. First, *gBRCA* patients with early-stage breast cancer have a good prognosis and, fortunately, distant metastasis is infrequent^38,39^. Of the 791 *gBRCA* early stage breast cancer patients that were followed longitudinally at MDACC, ~15% developed a distant recurrence, and the overall frequency of brain metastasis in the total *gBRCA* cohort was ~5%. As a result, our single-institution cohort of *gBRCA* patients with distantly recurrent breast cancer is small, though it is larger/comparable to those from previous reports. Second, the true incidence of brain metastasis is unknown since routine brain MRI screening is not recommended and is typically reserved for symptomatic patients. Thus, the estimates we provide almost certainly underestimate the true incidence. Third, though the clinical genetics database at MDACC is prospectively maintained and routinely updated, our study is retrospective in its scope and there are many uncontrolled factors including date of *gBRCA* testing, receipt and type of chemotherapy, and length of follow-up. A minor fraction of patients in all groups were lost to follow-up. Nonetheless, the strengths of our study include its use of a single, large database with comprehensive clinical annotation and the manual verification of all clinical data reported. The MDACC genetics clinic is an active and longitudinal program, reflected by the ~9-year median length of follow-up for *gBRCA* patients. As a result, we believe the data provided in this report are representative of the larger *gBRCA* population.

In conclusion, brain metastasis is a similarly frequent event among *gBRCA1* patients and *gBRCA* noncarriers with recurrent TNBC. In a competitive risk analysis from the time of primary breast cancer diagnosis, the 5-yr cumulative incidence of brain metastasis among patients with recurrent breast cancer was 38.8% for *gBRCA1* patients and 33.3% for noncarriers with TNBC (*P* = 0.26). Interestingly, studies of the molecular features of paired primary breast cancer/brain metastasis specimens, most of which are *BRCA1/2* wild-type, suggest that breast cancer cells capable of CNS propagation have increased homologous recombination deficiency, a characteristic intrinsic to *BRCA1/2*-deficient tumors. The therapeutic efficacy of PARP inhibitors for the treatment or prevention of CNS recurrence in *BRCA*-mutant (hereditary or somatic) or *BRCA* wild-type breast cancer is largely unknown, though the currently approved PARP inhibitors are unlikely to mediate durable CNS responses as monotherapy. Our study aligns with prior reports and underscores the importance of improving the prevention and treatment of brain metastasis in patients with recurrent TNBC, both in *gBRCA1* carriers and noncarriers.

## Methods

### Patient population and data collection

Approval was obtained from the institutional review board at UT MD Anderson Cancer Center (MDACC, approval no. PA18-0386). A waiver of consent was obtained to ensure ethical standards of data use due to the retrospective nature of the study. To determine the incidence of brain metastasis among *gBRCA* patients and *gBRCA* noncarriers, we queried an IRB-approved, prospectively maintained electronic database that includes patients referred to the MDACC clinical cancer genetics program who underwent *gBRCA* testing. Patients who test positive for various germline mutations or negative (noncarriers) are included in the database. We identified patients within the database with stage I-III invasive breast cancer who were evaluated at MDACC between 2000-2017 and who tested positive for hereditary mutations in *BRCA1* or *BRCA2* and assessed for disease recurrence and parenchymal brain metastasis. Patients with variants of uncertain significance in *gBRCA* were excluded from the analysis. Patients with leptomeningeal disease (LMD) were considered to have distant metastasis but were not included in our parenchymal brain metastasis cohort unless they had disease in both sites. The comparator group included noncarrier patients diagnosed with stage I–III TNBC who experienced subsequent distant metastasis. Clinical data from this cohort were extracted from the same database. The number of noncarrier TNBC patients with stage I-III invasive breast cancer between 2000-2017 within the database was too large for manual confirmation of clinical variables and so we limited the analysis to the 182 patients with distant metastasis.

Data elements collected included: patient demographics [sex, date of birth, and race (self-reported at time of new patient registration)], date of primary breast cancer diagnosis, date of last known follow-up, date of death if applicable (abstracted from chart or using the US Social Security Death Index), presence or absence of a second primary breast malignancy, *BRCA* status, date of *BRCA* testing, and the specific *BRCA* genetic alteration^40^. Disease characteristics collected included: receptor subtype (estrogen receptor [ER]/progesterone receptor [PR]/human epidermal growth factor receptor 2 [HER2]), anatomic stage (including T and N staging) at diagnosis, histologic subtype, tumor grade. Treatment characteristics collected included: type of surgery for primary breast cancer, chemotherapy delivered for primary breast cancer, distant recurrence and date if applicable, location of distant recurrence, and brain metastasis and date of detection if applicable. Brain metastasis characteristics and the treatment administered for the initial brain metastases were also abstracted from patients’ charts. Patients with Stage IV de novo metastatic disease were excluded.

Tumors were considered triple negative if both ER and PR were negative (<10% tumor staining) and HER2 was considered non-amplified. HER2 status was assessed by immunohistochemistry or fluorescence in situ hybridization when indicated and considered positive or negative on the basis of institutional cutoffs and guidelines that were current at the time of diagnosis. For tumor grade, composite histologic grade was used when available and if unavailable, nuclear grade was used. For patients with a second primary breast cancer, the most recent primary breast cancer was used in the analysis.

### Statistical analyses

Continuous variables are reported as mean or median (and range or interquartile range [IQR]). The distribution of each categorical variable is summarized in terms of its frequency and percentage. Distant metastasis-free survival was defined as the time from primary breast cancer diagnosis to distant metastasis (bone, liver, lung, CNS parenchyma, non-regional lymph node, etc.) or death from any cause. Patients still alive at the time of analysis were censored at their last date of follow-up. Time to brain metastasis was computed from the date of diagnosis of the primary breast cancer to the date of detection of brain metastasis on imaging or last follow-up. The cumulative incidence of brain metastases was estimated by Fine-Gray competing risk approach, considering death as a competing risk^41^. Among patients who developed brain metastasis, survival following brain metastasis was computed from the date of detection of brain metastasis to the date of death or last follow-up. Categorical data were compared using the Χ^2^ or Fisher’s exact test. Continuous data were compared using Wilcoxon rank sum test (two groups) or Kruskal–Wallis test (three groups). All tests were two-sided and *P* values < 0.05 were considered statistically significant as exploratory analyses. We used the Kaplan–Meier method to estimate survival and compared survival curves using the log-rank test. Computations were carried out using SAS version 9.4 and R 4.0.2 and GraphPad Prism 8.

### Reporting summary

Further information on research design is available in the Nature Research Reporting Summary linked to this article.

## Supplementary information

SUPPLEMENTAL MATERIAL

Reporting Summary

## Data Availability

The data that support the findings of this study are available from the corresponding author, upon reasonable request.
