# Supplementary material for: Incidence and impact of brain metastasis in patients with hereditary BRCA1 or BRCA2 mutated invasive breast cancer
Source: NPJ Breast Cancer. 2022 Apr 7;8:46. doi: 10.1038/s41523-022-00407-z (PMC8990006; doi:10.1038/s41523-022-00407-z)
Supplement: Supplementary file 1 — SUPPLEMENTAL MATERIAL [file 41523_2022_407_MOESM1_ESM.pdf]

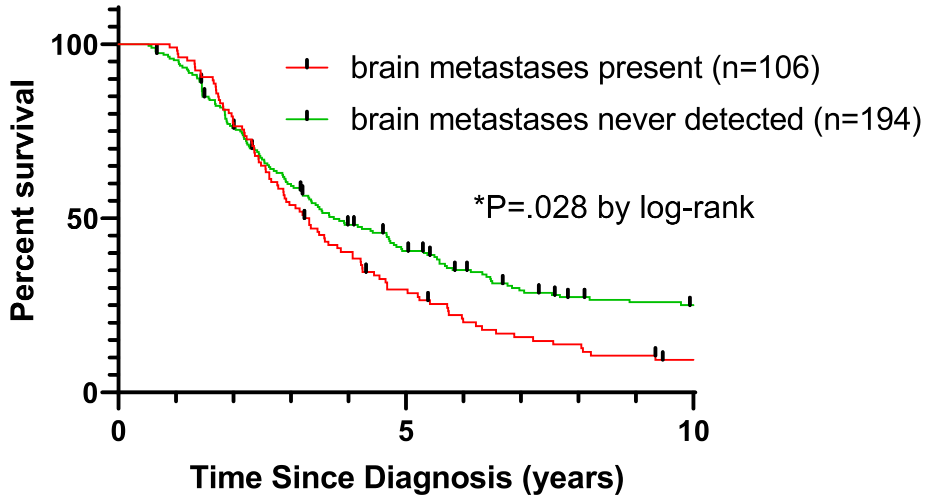

Number at risk:

yes brain met	*106*	31	8
no brain met	*194*	71	34

Supplemental Figure 1. Overall survival from the time of diagnosis for patients with recurrent breast cancer stratified by the presence or absence of brain metastases at any time during the disease course.

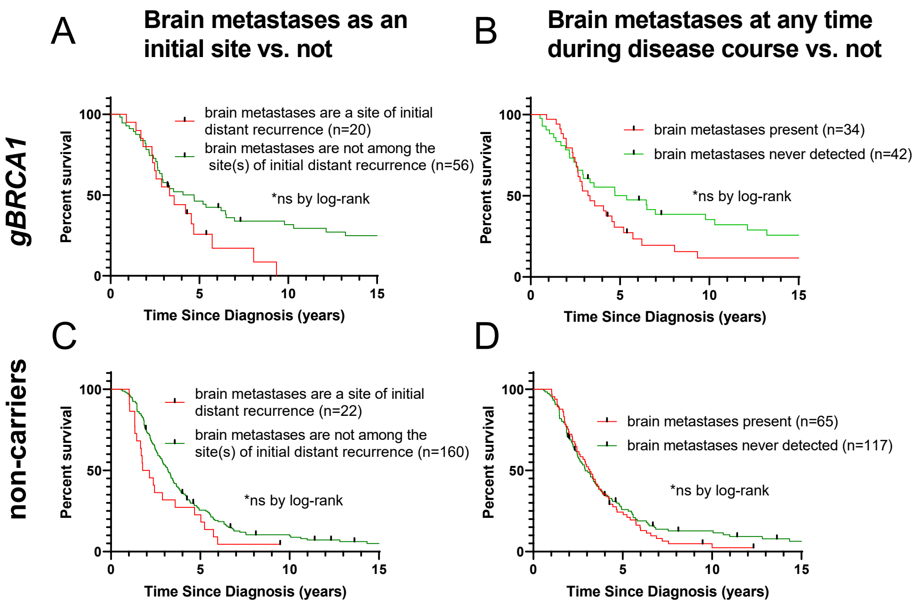

Supplemental Figure 2. Overall survival from the time of diagnosis for patients with recurrent breast cancer. In (A) and (B) are *gBRCA1* patients stratified by brain metastases as an initial site versus not and brain metastases detected at any time during the disease course versus not, respectively. In (C) and (D) are noncarrier patients with triple negative breast cancer stratified by brain metastases as an initial site versus not and brain metastases detected at any time during the disease course versus not.

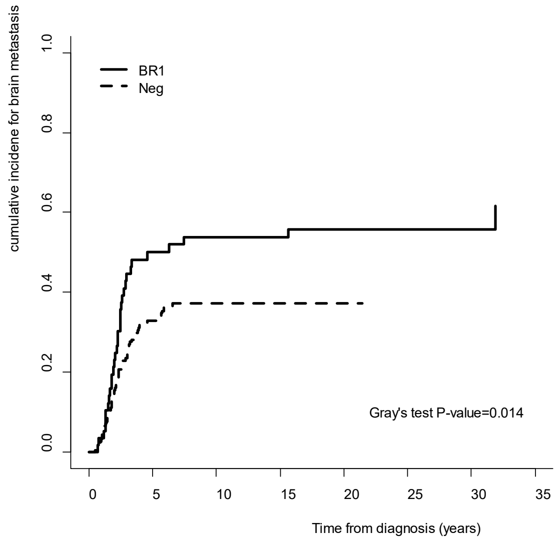

Supplemental Figure 3. Estimates of the cumulative incidence of brain metastasis are shown for *gBRCA1* patients with exclusively triple negative primary tumors (n=59) and noncarriers with triple negative breast cancer (n=182) diagnosed with stage I-III disease with subsequent distant recurrence.

Supplementary Table 1a. The association between brain metastasis and other clinical factors				
covariate	levels	yes brain mets	no brain mets	Fisher's Exact Test P-value (2-Tail)
Race	ASIAN/PACIFIC ISL	3 (27.3%)	8 (72.7%)	0.572
	BLACK	14 (30.4%)	32 (69.6%)	.
	HISPANIC	17 (45.9%)	20 (54.1%)	.
	WHITE	64 (40.3%)	95 (59.7%)	.
	OTHER	1 (20%)	4 (80%)	.
Breast ca subtype	HER2-positive	.(.%)	3 (100%)	0.0481
	HR neg/HER2 unk	1 (16.7%)	5 (83.3%)	.
	HR-positive/HER neg or unk	1 (10%)	9 (90%)	.
	TNBC	97 (41.3%)	138 (58.7%)	.
Second primary breast ca	no	96 (40%)	144 (60%)	0.0759
	yes	3 (16.7%)	15 (83.3%)	.
Cancer stage	I	13 (38.2%)	21 (61.8%)	0.1114
	II	54 (45%)	66 (55%)	.
	III	32 (31.4%)	70 (68.6%)	.
Tumor grade	I	. (.%)	5 (100%)	0.0356
	II	7 (23.3%)	23 (76.7%)	.
	III	87 (40.8%)	126 (59.2%)	.
Tumor histology	IDC	91 (38.9%)	143 (61.1%)	0.9314
	ILC	2 (40%)	3 (60%)	.
	other	6 (33.3%)	12 (66.7%)	.
Surgery for primary tumor	Mastectomy	69 (40.8%)	100 (59.2%)	0.6384
	Partialmastectomy	29 (35.4%)	53 (64.6%)	.
	ALND	1 (50%)	1 (50%)	.
Chemotherapy timing	adj	27 (34.2%)	52 (65.8%)	0.1957
	both	8 (34.8%)	15 (65.2%)	.
	neo	60 (43.8%)	77 (56.2%)	.
	none	4 (21.1%)	15 (78.9%)	.

Adjuvant radiation	no	22 (26.8%)	60 (73.2%)	0.0088
	yes	77 (44.5%)	96 (55.5%)	.
Group	gBRCA1	34 (44.7%)	42 (55.3%)	0.2064
	non-carrier	65 (35.7%)	117 (64.3%)	.

Supplementary Table 1b.				
The association between brain metastasis and other clinical factors				
covariate	brain_mets_cat	n	Mean+/-std, median(interquartile)	Wilcoxon rank sum test p-value
Age	no	159	46.03 +/- 10.98, 44.66 (37.79, 53.65)	0.1508
	yes	99	44.09 +/- 11.03, 43.04 (35.96, 52.84)	.

Supplementary Table 2				
Multivariable logistic regression of potential clinical factors in brain metastasis for gBRCA1 patients with TNBC and noncarriers with TNBC patients				
Effect	Point Estimate	95% Wald Confidence Limits		p-value
Tumor grade (III vs. I/II)	2.37	0.944	5.946	0.066
Presence of a second primary tumor	0.255	0.053	1.217	0.087
Adj radiation (yes vs. no)	2.268	1.243	4.139	0.008
