# Supplementary material for: Incidence and impact of brain metastasis in patients with hereditary BRCA1 or BRCA2 mutated invasive breast cancer
Source: NPJ Breast Cancer. 2022 Apr 7;8:46. doi: 10.1038/s41523-022-00407-z (PMC8990006; doi:10.1038/s41523-022-00407-z)
Supplement: Supplementary file 2 — Reporting Summary [file 41523_2022_407_MOESM2_ESM.pdf]

nature portfolio

Corresponding author(s): Nuhad K Ibrahim

Last updated by author(s): Jan 11, 2022

Reporting Summary

Nature Portfolio wishes to improve the reproducibility of the work that we publish. This form provides structure for consistency and transparency in reporting. For further information on Nature Portfolio policies, see our Editorial Policies and the Editorial Policy Checklist.

Statistics

For all statistical analyses, confirm that the following items are present in the figure legend, table legend, main text, or Methods section.

n/a	Confirmed	
☐	☒	The exact sample size (n) for each experimental group/condition, given as a discrete number and unit of measurement
☒	☐	A statement on whether measurements were taken from distinct samples or whether the same sample was measured repeatedly
☐	☒	The statistical test(s) used AND whether they are one- or two-sided<br>*Only common tests should be described solely by name; describe more complex techniques in the Methods section.*
☐	☒	A description of all covariates tested
☐	☒	A description of any assumptions or corrections, such as tests of normality and adjustment for multiple comparisons
☐	☒	A full description of the statistical parameters including central tendency (e.g. means) or other basic estimates (e.g. regression coefficient) AND variation (e.g. standard deviation) or associated estimates of uncertainty (e.g. confidence intervals)
☐	☒	For null hypothesis testing, the test statistic (e.g. F, t, r) with confidence intervals, effect sizes, degrees of freedom and P value noted<br>*Give P values as exact values whenever suitable.*
☒	☐	For Bayesian analysis, information on the choice of priors and Markov chain Monte Carlo settings
☒	☐	For hierarchical and complex designs, identification of the appropriate level for tests and full reporting of outcomes
☒	☐	Estimates of effect sizes (e.g. Cohen's d, Pearson's r), indicating how they were calculated

Our web collection on statistics for biologists contains articles on many of the points above.

Software and code

Policy information about availability of computer code

Data collection	Categorical data were compared using the X2 or Fisher's exact test. Continuous data were compared using Wilcoxon rank sum test (two groups) or Kruskal Wallis test (three groups). All tests were two-sided and P values < .05 were considered statistically significant as exploratory analyses. We used the Kaplan-Meier method to estimate survival and compared survival curves using the log-rank test.
Data analysis	Computations were carried out using SAS version 9.4 and R 4.0.2 and GraphPad Prism 8.

For manuscripts utilizing custom algorithms or software that are central to the research but not yet described in published literature, software must be made available to editors and reviewers. We strongly encourage code deposition in a community repository (e.g. GitHub). See the Nature Portfolio guidelines for submitting code & software for further information.

Data

Policy information about availability of data

All manuscripts must include a data availability statement. This statement should provide the following information, where applicable:

- Accession codes, unique identifiers, or web links for publicly available datasets
- A description of any restrictions on data availability
- For clinical datasets or third party data, please ensure that the statement adheres to our policy

The data that support the findings of this study are available from the corresponding author, upon reasonable request.

Field-specific reporting

Please select the one below that is the best fit for your research. If you are not sure, read the appropriate sections before making your selection.

☒ Life sciences ☐ Behavioural & social sciences ☐ Ecological, evolutionary & environmental sciences

For a reference copy of the document with all sections, see nature.com/documents/nr-reporting-summary-flat.pdf

Life sciences study design

All studies must disclose on these points even when the disclosure is negative.

Sample size	we identified 473 patients with gBRCA1 and 318 patients with gBRCA2 who were evaluated for stage I-III breast cancer at MDACC and who were captured in the clinical cancer genetics database.
Data exclusions	Patients with variants of uncertain significance in gBRCA were excluded from the analysis.
Replication	this is a retrospective study design with clinical data anaysis
Randomization	not applicable
Blinding	not applicable

Reporting for specific materials, systems and methods

We require information from authors about some types of materials, experimental systems and methods used in many studies. Here, indicate whether each material, system or method listed is relevant to your study. If you are not sure if a list item applies to your research, read the appropriate section before selecting a response.

Materials & experimental systems

n/a	Involved in the study	
☒	☐	Antibodies
☒	☐	Eukaryotic cell lines
☒	☐	Palaeontology and archaeology
☒	☐	Animals and other organisms
☒	☐	Human research participants
☐	☒	Clinical data
☒	☐	Dual use research of concern

Methods

n/a	Involved in the study	
☒	☐	ChIP-seq
☒	☐	Flow cytometry
☒	☐	MRI-based neuroimaging

Clinical data

Policy information about clinical studies

All manuscripts should comply with the ICMJE guidelines for publication of clinical research and a completed CONSORT checklist must be included with all submissions.

Clinical trial registration	not applicable
Study protocol	Approval was obtained from the institutional review board at UT MD Anderson Cancer Center (MDACC, approval no. PA18-0386).
Data collection	Between 2000 and 2017, we identified 473 patients with gBRCA1 and 318 patients with gBRCA2 who were evaluated for stage I-III breast cancer at MDACC and who were captured in the clinical cancer genetics database.
Outcomes	determine the incidence of brain metastasis in gBRCA patients presenting with early stage breast cancer. We also sought to determine the impact of brain metastasis on survival in patients with gBRCA and noncarriers with recurrent metastatic breast cancer.
